# The struggle for the social: rejecting the false separation of 'social' worlds in mental health spaces

**DOI:** 10.1007/s00127-023-02510-3

**Published:** 2023-07-03

**Authors:** Rochelle A. Burgess

**Affiliations:** Institute for Global Health, 30 Guilford Street, London, WC1N 1EH UK

**Keywords:** Social world, Intersectionality, Socio-political economy, Knowledge practices

## Abstract

How are we to best grapple with the notion of the Social in mental health landscapes? This piece of speculative work explores a series of tensions that emerge in our attempt to contemplate, engage with, and address the social in mental health spaces. First, I will explore the tensions created by disciplinary demands for specialisation, questioning the value of this with regard to treating social and emotional bodies which continually reject such fragmentation. This line of inquiry then leads to reflection on the value of a social topology—enabled through the application of intersectionality principles, Black Sociological analytical frameworks, including the worldview approach, and societal psychological perspectives on knowledge and action. I argue the possibilities in actioning these approaches emerge through the application of a social-political economy of mental health, that holds the complexity presented by the totality of social life as it potentially relates to mental health. The piece seeks to advance a space of thinking on how we transition global mental health projects to be more effectively situated in a needed commitment for social justice as a remedy and repair to broken social worlds.

## Introduction

Towards the end of my doctorate, there was a moment of clarity where I finally understood why my peers studying health in the social sciences could not see what I saw. It was during a session where I was describing my hope for the future of global health interventions. I was attempting to describe the models of care needed to meet the voiced needs of women living through adversity.

Following my presentation, a colleague approached me and said something along these lines:

*“Well to me, your interests and the interests of women themselves, are rooted in the socio-structural, and it seems that current services are designed with more interest in the socio-relational dynamics of wellbeing—you are asking us to focus more on the former, in response to an over emphasis on the latter”.*

My immediate response was one of confusion. I could not see what was gained from approaching the social as two separate dimensions. As Black woman of Caribbean heritage, I was all too familiar with my inability to separate my experience into different social categories. I felt, in a similar way to my research participants, that my agency was always constrained by social dynamics that were simultaneously structural, relational economic, and political. This moment was the beginning of a line of questioning that has shaped my work for the last decade. It has always appeared to me, a falsehood, for scholarship to separate into component parts what people themselves view as enmeshed, entrenched, and indivisible aspects of their lives. I have conducted qualitative and ethnographically informed research on community mental health for many years. The narratives I encountered were never ‘socio-structural, *or* socio-relational, *or* socio-political’. They were all these things, all at once. And despite the criticism that siloed approaches have faced [1], we continue to exist in, and advance, platforms where systems responsible for promotion of good health cannot meet complex social beings in their complexity.

Professionally, I have come of age alongside the *movement for global mental health (GMH)*, who, for better or worse, exists in the wake of wider psychological and psychiatric discourses that fall victim to these separations [2]. This problem is compounded by the fact that GMH is also working within the equally problematic wake of colonialism, racism, and its ramifications on the policy landscape of global health more widely domain [3]. The consequence of overlooking these wakes and what they produce has been the creation of necessary but insufficient treatment and knowledge production spaces which, while increasing access to support in some ways, also minimise the importance the damaging social worlds where bodies live. This outcome is crystalised in the closing report of former UN Special Rappator, Puras in 2021 where he frankly states that the global mental health response is:

*“Not focused on how poverty and social injustice can produce mental distress. The focus has been on the burden and cost of mental health disorders. That is not consistent with a human rights-based approach and has been shown to be methodologically flawed. The focus remains on individual rather than systemic change as a means of tackling poverty and oppression.” * [4].

It is worth asking the question, what do we gain and lose, from our separation of the social within our current modes of engagement in the lives of others in global mental health? And how can we respond to the gaps that our current engagement creates in our research and practice?

This piece is a speculative work, seeking to explore a series of tensions that emerge in our attempt to contemplate, engage with, and address the social in mental health spaces. First, I will explore the tensions created by disciplinary demands for specialisation, questioning the value of this with regards to treating social and emotional bodies which continually rejects such fragmentation. I reflect on the case study of a woman encountered during my doctoral research in 2010 which has remained with me for many years, originally published in 2016 (See [5]). I suggest the need for, and tensions in applying a ‘unifying theory’ of the social—or social topology—enabled through the application of (1) intersectionality principles, (2) the worldview approach; a phenomenological framework rooted in Black sociological perspectives, and (3) societal psychological perspectives on knowledge and action. I then reflect on the possibilities in actioning a social topology approach through the use of a social-political economy of mental health, which seeks to hold the totality of ‘social life’ as it potentially relates to mental health. The aim of this piece is not to generate solutions, but to advance a space for thinking through how we may finally transition global mental health projects to be more effectively situated in a commitment to social justice as a remedy for broken social worlds.

### The body must testify: tensions in acknowledging the reality of the social

Psy-knowledge production is rooted in and sustained by, an acknowledgement of relationships between the biological and the psychological. In his Archaeology of mental health [6], Foucault draws our attention to the ways in which psychiatry in particular must obtain/sustain its status within the health sciences through performance that draws attention to, and mirrors the logics of, biological and medical sciences. What necessitates this performativity is rooted the wider social contexts of this period. Psychiatry in its early years was a medical specialisation that was linked to and judged by the same stigmas that patients carried (though of course, patients endured the blunt end of these social rejections). For psychiatry to shed these judgements and obtain markings of respectability required a systemisation and mechanisation of its approaches (see [7]). This has resulted in a psychiatric practice that runs in parallel with biological sciences—with various sub-disciplines and specialisations which focus on specific types of relationships between the brain and body (such as neuropsychiatry), life-stages (adolescence and older age), or life experiences (addiction).

Therefore, the longest standing critiques facing mental health practice reside in its capacity to appropriately acknowledge the social dimensions of patients’ lives. For example, within the 13 faculties/specialisations listed on the Royal College of Psychiatrists website,^1^ only one of them makes explicit mention to the social: *Rehabilitation and social psychiatry*—which is focused on supporting recovery and reintegration of people with long-term, complex psychiatric conditions back into the community. Social science-based critiques of psychiatric practice are typically anchored to demands for the social—seeking to illuminate that much of what counts as a ‘good life’ are inseparable from social factors [9]. This has been a necessary reaction and has driven many crucial adaptations within approaches to treatment and care in the field. However, despite the advance of discourses that show appreciation for the social in relation to mental health, evidenced most recently by wide support for ‘social determinants’ approaches, ongoing critiques suggest that we have not gone far enough. Why is this the case? I feel that our inability to take the social seriously, is created by two separate but related processes: the fragmentation of the social, which I explore below and, power–knowledge relationships which I explore in the following section.

First, the fragmentation of the social has been validated within social science engagement. Scholars in the social sciences, particularly quantitative social sciences, typically negotiate within the space of the social by breaking it into constituent parts through the creation of typologies (see Table 1). The aim of typologies is to divide, and the division is felt to enable analytic tasks and refining of concepts, as well as the creation of categories for measurement, classification, and sorting of cases [9]. However, there are two challenges with such an approach, which also direct us to my second process of knowledge–power relationships. First, the division into categories does not happen in the absence of simultaneous and often underacknowledged hierarchisation or value around these categories, as they lead to specialisations. In the process of separating, here too, specialisations mean that certain aspects of the social gain more importance within each discipline. Political science prioritises the domain of socio-political, economics, the socio-economic, and so on. As these ideas are picked up within the health landscape, too quickly aspects of social life move beyond the responsibility or specialisation of practitioners, or social categories are focused by individual actors within multi-disciplinary teams.

**Table 1:** A typology of the social

Dimension	Definition
Socio-relational	Voluntary or interpersonal relationships between actors, groups, and organisations that exist within social structures
Socio-political	Relating to or involving a combination of social and political factors (social structures that are shaped by political policy, practices, and social behaviours)
Socio-economic	Relating to or concerned with the interaction of social and economic factors (social structures that are shaped by economic policy, practices, and behaviours)
Socio-structural	Relating to the social structures of society, often linked to five levels of analysis from western European sociological scholarship—the structure of the family, law, religion, economy, and class

To illustrate what this means in practice I want to reflect on the case of a patient I uncounted during ethnographic research in 2010. In Box 1, I present the case of patient S, which I feel illuminates that the consequence of our current approach to manifestations of the social, even within a multi-disciplinary team. For Patient S, the separation leads to a separation of the person. Rather than being seen as a part of a unified whole, each aspect of her world is partitioned off, handled by a different department approaching her in slightly different ways. A doctor embedded within the social welfare department, who is responsible for opening gateways to socio-economic supports—and denies them. A psychologist who is willing to treat her psychiatric concern only if her ‘obsession’ with her economic realities (i.e., her desire for a welfare grant) can be left alone. At the worst, her demands for a social response are medicalised and identified as malingering. Her insistence that the entirety of personhood be seen, and be treated, is approached with frustration, and in some instances outright rejection, which occurs at the intersections of multiple planes of social life.

**Figure:**
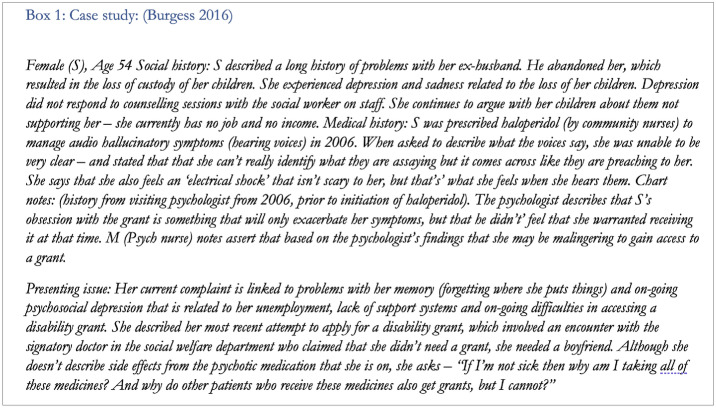

As is shown in the case study, S’s embodied experience of her social world is not differentiated. In her reality, the socio-economic directly shapes the socio-relational and so on. When we approach bodies in ways that are shaped by perspectives rooted to a differentiated typology of the social, these social categories are stripped of their intersecting meanings. We end up treating social factors as empty categories, overlooking the processes which link specific and multiple categories, to specific bodies across and through time.

In exploring possible opportunities for moving past these tensions around the social, I have been drawn to Deleuze’s [10] reflections on Foucault’s archaeology of knowledge. Foucault’s archaeology involves a process of grappling with the origins of a concept or idea (or knowledge) manifested as ‘statements’ in our speech and writing and discourse. In his approach, practices and concepts often taken for granted or assumed are interrogated to investigate their roots. Deleuze suggests that this archaeology enables us to think about the spaces that exist *around* discourses or statements. These thought processes allow us to observe how claims about what is included our excluded from our conceptualization of ideas (i.e., discourse) only gain their meaning and value through context or situated realities connected to the idea. In this essence, when the idea of the social is invoked, it establishes relationships to subjects, objects, and concepts which create and mean things to serve specific ends.

A rapid exploration of some common typologies of the social via their dictionary definitions results in a series slightly repetitive statements outlined in Table 1. Viewing these together, we are confronted with repetitions bringing each term closer to another—nearly identical formations which shift depending on the contexts in which they are uttered and the aims the specific term serves. As part of their separation, we establish pathways for relationships to different sets of actors each holding different sets of responsibility for action.

In the repetition and slippage of their boundaries, we begin to see that the social is likely better viewed as the sum of its parts. For example, the socio-relational refers to social structures, like when one examines the impact of colonialism on relationships within family structures. Deleuze suggests that this repetition is only possible, because there is the presence of the single definition that precedes these statements which exists somewhere in our collective consciousness. This alludes to the possibility of a core idea of the ‘social’ contained within the statements in Table 1 and intimates that we may approach the ‘social’ as *topological,* rather than typological concept. Topologies are rooted in mathematics and push us towards recognising that within a given entity exists potentially infinite combinations and iterations that can emerge from the original. Crucially, even as a topological shape shifts and transforms into alternative iterations of the original, its core properties are maintained and preserved. Thus, topologies exist as structures which allow for the definition of continuous formations and reformations of subspaces, and ultimately multiplicities of a central notion. With topologies, even as things change, they maintain a core; they stay the same [11].

If we view the social as a topological space, then it assumes that varied social realities entwine and transform each other, rather than work independently. I would like us to view ‘the social’ in relation to mental health as a topological space: a plane holding the totality of what it means to *be* in this world. This is beyond static categories and structures, but rather attunes to what specific formation of the social is created through the interaction of these categories for people as they exist in, move through, create, resist, reject, and transform those categories. In this essence, the social is the unending dialogue made between people, places, space, history, and identity—a social landscape of our lives. A topological space is akin to what Black Feminist scholars articulate as intersectional orientations which conceive of social categories as constantly changing, fluid, and being created and re-created by power relationships [12]. By orienting ourselves to how bodies are located within the *totality of the social space*, we can draw our attention more firmly to what the social produces in the lives of others, in ways that are meaningful beyond biomedical outcomes. The next challenge emerges in what we do practically capture and how we act in relation to topological spaces.

### Responding to living evidence: using an intersectional socio-political economy to shift global mental health practice

The movement for global mental health is at a crossroads, because our tools do not match the demands of people building a life within often impossibly complex social worlds. Many practitioner and researcher attempts to take the social world seriously are insufficient because they often engage in a process of ‘unknowing’, where they see only pieces or partial categories, rather than the complexity bodies carry. As argued previously, a social topology may help us to see the whole of the social, but it does not automatically disrupt knowledge hierarchies embedded within the global mental health landscape. As Deleuze’s notes: *“But one also sees, then, how certain multiplicities, and formations direct the knowledge… haunting them not towards epistemological thresholds, but in very different thresholds.”*([10], p, emphasis added)*.* In patient S’s case study and in testimonies of many patients and actors across mental health landscapes, we see that a shared platform is not always possible. Reasons behind this are well explored within Social Representations research (SR), a social psychological theory of knowledge and action. SR perspectives view knowledge as linked to action through projects [13]. Each specific project is advanced over time, mediated by context as well as the power held by other knowers who come to work towards a shared issue. As multiple actors come together around a supposed shared goal, the terrain is not always smooth, as each group of actors advance their own interests, mobilising various forms of symbolic, structural, or material power at their disposal [14]. As such, when a project demands the application of various knowledge(s), the knowledge that is likely prioritised is always determined by how certain groups leverage their own power in service of their interpretation of what ‘should’ happen.

Through this lens, we can understand how collective action towards a shared goal (like those directed by multi-disciplinary teams) struggles to draw on all forms of knowledge equally. Difficulties emerge in staying with the complexity of the social, as knowledge mobilised by each actor drives others in specific directions with different consequences. For example, it is widely accepted that the distribution of power across multi-disciplinary teams is uneven [15], and non-medical actors struggle to lead in these spaces. That is even more the case for patients. For S, family difficulties and depression may require support from a psychologist. Yet, a psychologist whose main project is the restoration of psychological and emotional balance somehow pulls the patient away from her own project anchored to addressing the worries created by how those relationships are shaped by economic life, gender relations, and social norms. Her knowledge of these realities cannot compete with or find paths for action in a space where the project and action interests of others are single-issue focused (psychological or socio-structural). To be supported with economic challenges requires travel to the social work office, pulling her into another threshold with different systems of operation, and other projects over which she has very little control. Elsewhere, when experts by experience are included in knowledge-generation and treatment activities, this too has been fraught with similar tensions around knowledge and power [16]. Ultimately lived experience knowledge is largely only valued when it is in service to the mainstream medical apparatus [17], and the breadth of the domain of mad studies is an illumination of the wider social realities and possibilities that are not seen within services [18].

How might we address this? Generations of anthropological scholarship have also been devoted to the articulation of social and cultural dimensions of mental health and the importance of services that respect these narratives (see [19–21] for Kirmayer’s work advancing culture and Kieser’s and Weaver’s work on idioms of distress). However, these frameworks can meet their own limits within mental health encounters, where a ceiling limits how far we can action alternative social knowledges beyond their ability to support medical practices or understandings of biomedical treatment. While this is of course important, using knowledge in this way does not centre the totality of the social. Take, for example, Kleinman’s explanatory models, a methodology devised to include lay understandings and definitions of health and illness within diagnostic frameworks. They are often celebrated as a mechanism for taking local knowledge seriously in successfully treating mental health challenges. However, applications of this method in clinical settings struggle to take seriously patient claims to knowledge when it demands action in realms beyond the biological, psychological, or relational. As seen in Dino’s and colleagues [22] reflections on the application of explanatory models in clinical practice, the priority and commitment remains to biological components of care, and cultural models which unseat that, remain difficult to hold as valid. In truth, the ‘social’ is still primarily seen as a mechanism to promote the uptake of biomedical care. Whether explicit or not, a hierarchy remains in how we value knowledges about mental health, and within this, our calls for better diagnosis and more effective treatment thus continues to prioritise knowledge systems used in biomedicine. The global mental health project remains owned by medicine. The primary actor remains a biomedical one, and the project that is mobilised for change, is a largely biomedical project too. Recent work of UK psychotherapist James Barns supports this view—asserting that the attempts of psychiatry to grapple with the social have not disrupted the allegiance to biomedical or specialist responses [18].

Furthermore, as global mental health continues to situate itself within addressing the needs of the majority world,^2^ part of advancing the complexity of the social must also acknolwedge that our dominant systems of knowledge drive logics of care rooted in Eurocentric perspectives of health and healing. Recent work by Jessica Horn [23] articulates that even when culturally adapted, current treatment approaches can be a poor fit due to its narratives and knowledge systems indigenous to the worldviews of majority people their suffering and their patterns of healing. Part of responding to these absences is an active effort to recentre historically excluded or minoritized thought [24]. In his writings on Black Sociologies, Carroll [25] draws attention to what he argues are core ways in which the African-centred world views also present a ‘unifying’ social theory rooted in historical spaces, places, and ways of knowing. These are different to and separate from those anchored to European anchored knowledge systems which work towards separation. He writes:

*“While the African world view prioritises an interconnected and interrelated reality that relies upon the immaterial aspects of reality to make sense of the lived experience and favours relations of the whole, the European worldview prioritises the separation of social reality, only utilizing that which can be apprehended with the five senses to validate and provide meaning for that which we engage through our lived experience” Carroll, 2014 pg 260.*

The ‘worldview’ cited above was established by African American scholar Vernon Dixon, who suggests that sense making of our world is filtered through a series of philosophical assumptions about cosmology (the universe), axiology (values), ontology (ways of being), and epistemology (knowledge). The first two are argued as central to African-centred thought (see [25]), and lead to a major difference in understanding that knowledge is acquired through interconnected experiences and linked to structures that exist beyond the five senses (or that which can be observed). Furthermore, the worldview has parallels with Societal Phenomenology, meaning that it still sits in relationship with, rather than outright rejection of the sensemaking processes within more critical aspects of European scholarship, with both suggesting that experiences gain meaning through personal and intersubjective experiences projected against both a concrete and metaphysical space where life is ‘lived’—the daily backdrop to our experiences [26].

If it is possible that the worldview promotes a more actionable plane for holding the multiplicities of the social, it is because by design the worldview approach resists the hierarchies of knowledge that underpin current models. The worldview sees and listens to the complexity of lives, because it is a paradigm that is oriented towards pluralism. In resisting the hierarchisation of not only the social, but more importantly, knowledges itself, it simultaneously orients itself towards an ethics and pragmatics of care that promotes a transition of the ownership of the mental health project to the patient and communities.

So what might this mean in practice? Within a worldview framework, responding to the case of Patient S would *centre* the inseparability of her poverty, distress, and familial struggles. The search for a remedy would demand that practitioners follow her self-defined project of interest and trigger a set of activities which look entirely different to what has been described in her reality thus far. By remedy and repair,^3^ I refer to the need to ‘make right’ the ruptures in her social world. This extends our focus to logics of care beyond treatment and towards social change. The role of the practitioner then becomes one that also seeks to promote opportunities for remedy and repair in much wider domains of the social world.

**Fig. 1:**
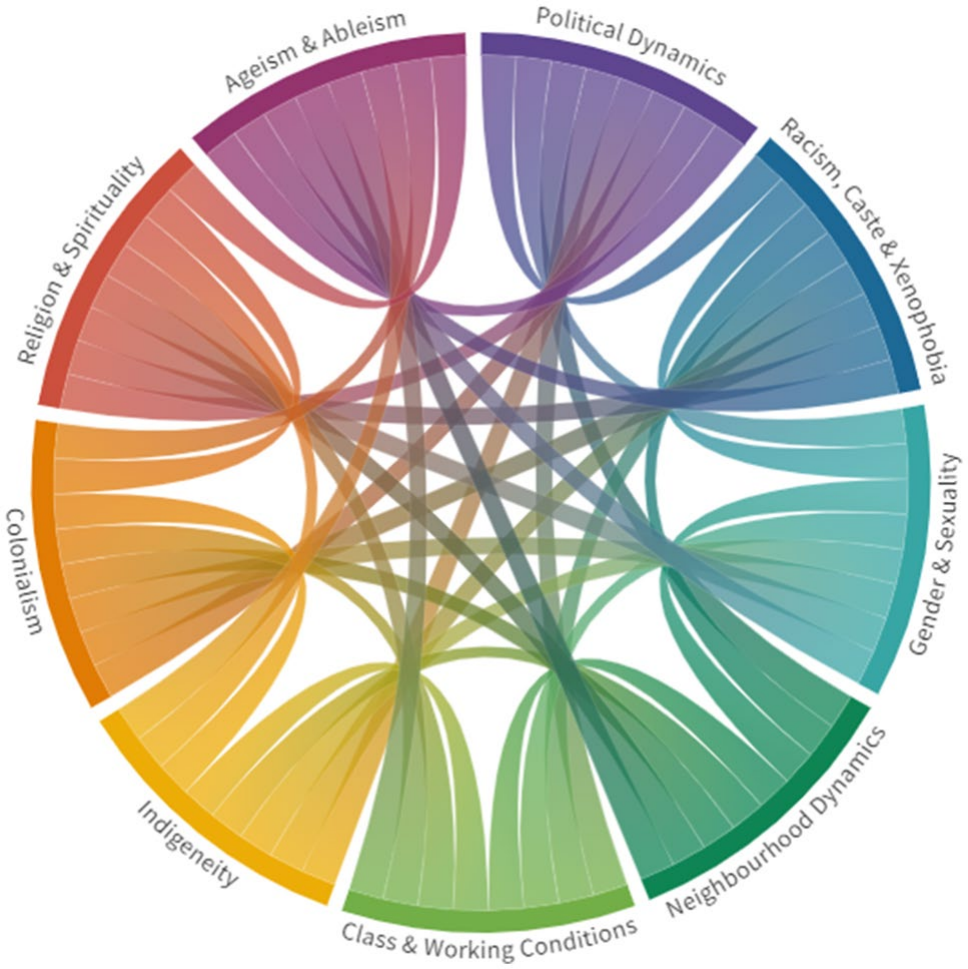
Visualising the worldview through a socio-political economy of global mental health [27]

To implement such logics of care, would reqiure tools that illuminate multiple dimensions of the social landscape able to guide patients and practitioners in dialogues which support a process where patients and medical actors of call kinds could to locate themselves to particular space within a social plane, established by the intersection of social. In Fig. 1, I present a proposed model for a socio-political economy of global mental health. This model seeks to hold together multiple aspects of the social world: political, economic, historical relational, and structural dimensions. Linked to political economic theory which draws our attention to the conditions with determine the patterning of health and illness [27], the model moves us beyond culture diagnosis, and draws us closer to key dimensions of social processes which determine everyday existence.

Critical to this model and its visualisation is the interweaving of these positionalities. The crossings and intersections are illuminated as each category meets another and produces new colours and new ways of being in the world. It becomes impossible to ignore that someone’s lived experience is driven by any and potentially all dimensions of the social world, if such a framework is presented. This figure makes tangible this intersection and could serve as a valuable platform for the holding of the complexity of the social that is required as part of practice, and research for social justice-oriented mental health practices.

The dimensions of the framework are purposefully broad. The precise content and meaning of each category would need to be defined within discursive spaces and shaped by cultural and contextual realities of locations. Because this framework is rooted in principles of intersectionality, the framework demands a flexibility that would allow it to change to match the dimensions of the social plane which make the most sense in a particular setting, to a particular group. However, this fluidity also reiterates the importance of ‘staying with the complexity’ of the social and provides a method that is attuned to the infinite possibilities of the social and delays the insertion of hierarchies or an automatic ‘giving in’ to the seduction of a single way to define or respond to the social in people’s lives.

While I lack the space to do so in this manuscript, future work would need to explore the translation of a figure like this into an actionable toolkit which provided clear examples of how to defining and work through each dimension.

## Conclusions

In an era inundated with call to arms around mental health in high and low resource settings alike, my request is this. When it comes to the social, efforts to hold its complexity must also be accompanied by a commitment to shifting the ownership of the global mental health (or any mental health) project to patients and people living through adversity. A social topology provides a pathway to achieve this, and promote a logic of care that provides remedy to many ills: including both the biological and social in its totality. This is ultimately, the establishment of a praxis of global mental health: which requires a radical rejection of the current pathways, and a commitment to centring ways of thinking and acting that are driven by the testimonies of bodies that the field seeks to support. Any other response means that mental health research and practice will find itself locked within a space unable to deliver justice, or take seriously what the social means for patients, communities, and people globally.

## Footnotes

1 See https://www.rcpsych.ac.uk/members/your-faculties [last accessed 29th April, 2023].

2 The majority world is used here to refer to the notion of the global majority, reflecting that historically marginalised groups actually make up the majority of the global population.

3 I am grateful to Prof. Kit Davis, for her suggestion of this metaphor within the context of my work.
